# Failure analysis and structural design improvement of support device for heavy-duty rack and pinion hydraulic actuator

**DOI:** 10.1038/s41598-026-46597-5

**Published:** 2026-04-18

**Authors:** Duo Zhang, Bingqian Luan, Fujian Chen, Xiangqian Zhu, Yudong Xie, Yi Wan, Shuai Ji, Chuanying Wang, Jin-Hwan Choi

**Affiliations:** 1 State Key Laboratory of Advanced Equipment and Technology for Metal Forming, Shandong University, Jinan, 250061 China; 2 Key Laboratory of High-Efficiency and Clean Mechanical Manufacture of MOE, School of Mechanical Engineering, Shandong University, Jinan, 250061 China; 3 JIER Machine-Tool Group Co., Ltd, Jinan, 250022 China; 4 Department of Mechanical Engineering, Kyunghee University, Yongin, South Korea

**Keywords:** Rack and pinion hydraulic actuator, Support device, Failure analysis, Finite element method, Design improvement, Engineering, Materials science

## Abstract

This study investigates failure analysis and design improvements for a linear rolling guide (LRG) support device in a heavy-duty rack and pinion hydraulic actuator. Premature crushing failure of the end rollers in the LRG support device was observed during prototype field testing. In order to identify the underlying causes, a high-fidelity finite element model of the rack and pinion hydraulic actuator and the LRG support device was established and subjected to numerical analysis. The results indicate that the LRG roller failure is intrinsically governed by non-uniform load distribution in both the longitudinal and transverse directions, leading to excessive local contact stresses at the end rollers, rather than by local material deficiencies or manufacturing defects. On this basis, two improved support schemes of sliding and track roller support devices were proposed. Comparative verification via finite element analysis demonstrates that both improved schemes can significantly reduce contact stress levels. The sliding support device achieves the lowest stress due to its substantially increased effective contact area, whereas the track roller support exhibits superior load distribution characteristics. This study provides a theoretical foundation and practical engineering reference for failure analysis and structural design of support devices in heavy-duty rack and pinion hydraulic actuators.

## Introduction

As a critical transmission and load-bearing component in large-scale stamping equipment, forming machinery, and special-purpose actuators, the rack and pinion hydraulic actuator plays a decisive role in overall system performance. Its operational stability directly governs the positioning accuracy, load-carrying capability, and service life of the equipment^1,2^. Linear rolling guide (LRG) provides effective vertical support and precise guidance, featuring high positioning accuracy and low static and dynamic friction coefficients^3–5^. Consequently, they are widely employed as rack support devices in engineering applications. However, under heavy-duty conditions, LRG is highly susceptible to uneven stress distribution among the rollers due to manufacturing tolerances, installation deviations, and the incompatibility between rack and support seat deformations^6^. Such extreme load non-uniformity can cause localized stresses in the end rollers to exceed the material’s allowable limits, ultimately resulting in accelerated roller wear or even surface crushing failure. Therefore, it is imperative to investigate the failure mechanisms of LRG support devices and refine the design of support structures for heavy-duty rack and pinion hydraulic actuator to enhance their load-bearing performance.

Regarding the mechanical behavior of rack and pinion transmission and support structures, Cao et al.^7^ derived the load distributions for individual gears within multi-set systems in self-elevating platforms under diverse operational scenarios. Tao et al.^8^ established a contact stiffness calculation model for roller guide pairs predicated on the Palmgren formula and rigid-body dynamics, thereby elucidating the contact mechanics between rollers and raceways. Chen et al.^3^ employed a micro-convex body group approach to simulate fatigue wear models for roller guides, facilitating an analysis of their underlying wear mechanisms. In recent years, concurrent with advancements in computational and numerical techniques, Finite Element Analysis (FEA) has become a cornerstone methodology for investigating complex contact problems. FEA not only reveals the mechanical behaviors associated with structural deformation from a macroscopic perspective^9,10^, but also enables the investigation of extreme loading conditions that are difficult to impose or reproduce experimentally, while significantly reducing the cost and risk associated with design iterations. Ahmed et al.^11^ examined the geometry and contact positions of rack and pinion systems at different rotation angles and employed FEA to analyze the stress and stiffness of offshore drilling platform jacking systems under extreme sea states. Gong et al.^12^ established an FEA model of the rack and pinion transmission system of the Three Gorges ship lock and evaluated gear bending stress under different wear levels. Subasi et al.^13^ proposed an adjustable locking bone plate integrating a rack and pinion fine-tuning mechanism, determining the maximum safe torque range for surgeons through mechanical failure FEA. Nicolalde et al.^14^ developed a material selection framework for automotive rack and pinion systems based on multi-criteria decision-making method, validating the mechanical feasibility of selected materials relative to lightweight and safety requirements via FEA. Gulisano et al.^15^ proposed an automated finite element–based approach for load-dependent contact analysis of complex gear geometries and applied it to rack and pinion contact stress evaluation in steering systems. Chang et al.^16^ established a dynamic FEA model for rack and pinion components in aircraft nose landing gear steering assemblies to analyze meshing processes and stress characteristics. Niu et al.^17^ proposed a novel pure rolling variable transmission ratio herringbone rack and pinion configuration for commercial vehicles and investigated its contact stress distribution using FEA. Sun et al.^18^ analyzed the dynamic characteristics of LRG in the support direction using FEA and validated the results through analytical solutions and experiments for CNC machine tool dynamics. Kwon et al.^19^ employed FEA to calculate deformation of LRG carriages induced by contact loads. Song et al.^20^ proposed an improved LRG design method and investigated the influence of roller profiles on stiffness and stress distribution through finite element simulations. Liu et al.^5^ constructed a ball combination interference model based on Hertz contact theory, accounting for both preload and various geometric errors, to analyze their impact on static load distribution and deformation in LRGs, with the model’s accuracy being corroborated through experimentation. Stosiak et al^21^ investigated the feasibility of replacing traditional steel with carbon fiber-wrapped composite materials and PET engineering plastics for manufacturing hydraulic cylinder barrels and end caps, combining FEA with experimental validation. Existing studies have predominantly focused on the rack pinion transmission pair or on standard LRG units, while investigations into the mechanical behavior of dedicated support systems for rack and pinion hydraulic actuators under high-load conditions remain limited. Specifically, systematic and targeted analyses of non-uniform roller load distribution, end-roller stress concentration mechanisms, and support structure optimization are still lacking. Furthermore, conventional designs largely rely on empirical formulas or simplified calculations, which are inadequate for accurately capturing the complex internal load transfer paths within supporting rollers and their underlying failure origins. This limitation further underscores the imperative for high-fidelity simulations and the structural design improvement of support devices.

Building upon the aforementioned research, this paper investigates a heavy-duty rack and pinion hydraulic actuator and its dedicated support device. To address the engineering failure characterized by non-uniform load distribution in the LRG support structure, which results in crushing failure of the end rollers, a comprehensive finite element model incorporating the actuator housing, the rack-pinion pair, and the LRG support device is developed using Abaqus 2022. The failure mechanisms of the LRG support device are systematically investigated based on this model. Subsequently, two improved schemes of sliding and track roller support devices are proposed. FEA are conducted to comparatively evaluate the contact stress, load distribution, and supporting forces across the three support devices under identical operating conditions, thereby verifying the effectiveness of the proposed improved support designs. Although the failure originates from a specific hydraulic actuator system, the mechanical mechanism and analysis framework identified in this study are applicable to a wide range of heavy-duty LRG systems subjected to eccentric loading.

## Failure description

**Fig. 1:**
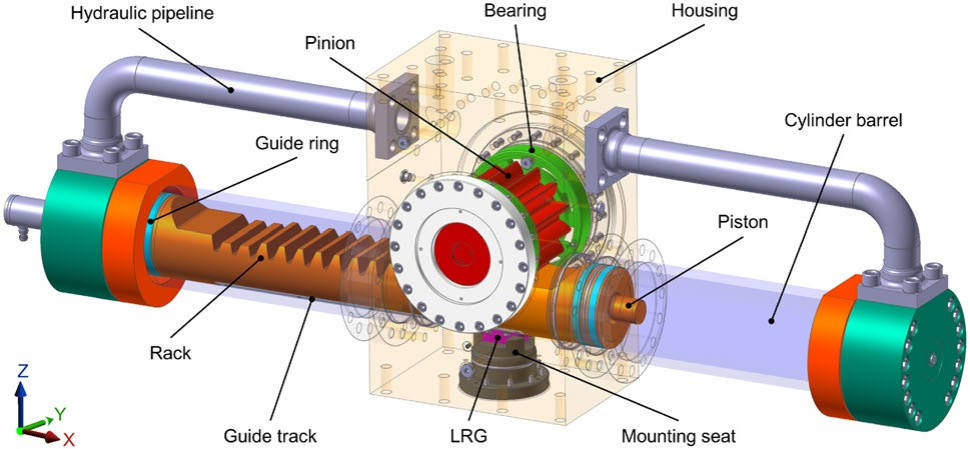
Geometric model of rack and pinion hydraulic actuator and its integrated support device.

This study focuses on the analysis of a practical heavy-duty rack and pinion hydraulic actuator and its dedicated support structure. A schematic illustration of the actuator and its support system is shown in Fig. 1. The rack and pinion hydraulic actuator primarily consist of the housing, rack and pinion pair, guide track, piston, guide rings, bearings, cylinder barrel and hydraulic pipelines. In this study, the X-axis is defined as the longitudinal direction along the rack motion, the Y-axis as the transverse direction, and the Z-axis as the vertical direction. The rack is integrally manufactured with the piston, and guide rings are arranged at both ends of the rack to provide auxiliary radial support and motion guidance.

**Fig. 2:**
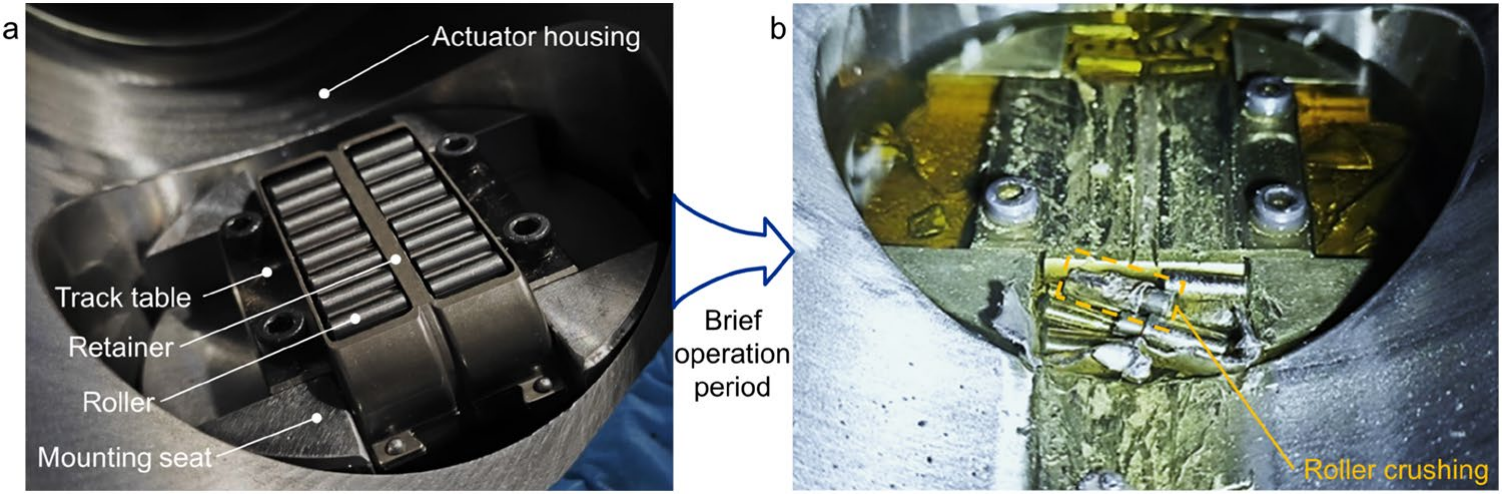
LRG support device. (**a**) Installation location of the LRG. (**b**) Roller crushing failure.

The LRG support device is affixed to a mounting seat at the base of the actuator housing and mainly consist of rollers, retainer and track table. The rollers are arranged in two parallel rows, with a total of 16 units, providing effective support during the reciprocating motion of the rack along the cylinder axis, thereby resisting rack bending deformation and carrying a portion of the radial load. The actual installation position of the support device within the rack and pinion hydraulic actuator is shown in Fig. 2a. Nevertheless, under nominal dynamic loading conditions, hydraulic oil leakage occurred after only several hundred reciprocating cycles of the rack and pinion hydraulic actuator. Subsequent disassembly of the actuator revealed crushing failure of the LRG rollers, as illustrated in Fig. 2b. The fragmented roller debris entered the hydraulic actuator, resulting in seal damage and ultimately causing oil leakage. Notably, the occurrence of roller crushing failure was far earlier than the designed fatigue life. The roller damage morphology, characterized by severe crushing and fragmentation, indicates overload contact rather than false brinelling, which typically presents as periodic indented grooves caused by micromovements in stationary bearings^22^. In addition, severe wear was observed on the guide rings mounted on the rack. This abnormal failure mode indicates that the current support structure of the heavy-duty rack and pinion hydraulic actuator suffers from improper design and component selection.

The premature crushing failure of the unilateral rollers of LRG support device, coupled with the severe wear of the guide rings, indicates significant localized load non-uniformity under actual operating conditions. Due to the combined effects of high load levels and long stroke operation, the rack is prone to flexural deformation, which generates a tilting moment primarily in the X-Z plane, with the moment vector oriented along the Y-axis. This moment is transmitted to the LRG support device, resulting in eccentric radial loading. Consequently, the radial reaction forces are unevenly distributed among the rollers, with the maximum load concentrated on the outermost rollers, causing the local stress to exceed the material yield limit and ultimately leading to quasi-static overload failure.

## Finite element analysis and failure mechanism investigation

### Establishment of finite element model

Finite element analysis is conducted to investigate the stress characteristics of the heavy-duty rack and pinion hydraulic actuator and LRG support device, and to validate the failure mechanisms of the support structure described in Section "Failure description". The finite element model of the actuator and LRG support device is established using HyperMesh 14.0. During the process of modeling, non-critical local features that exert minimal influence on the overall structural performance are appropriately simplified to enhance computational efficiency. The characteristic parameters of the actuator and LRG support device are summarized in Table 1.

**Table 1:** Characteristic parameters of actuator and LRG support device.

Parameter	Value
Pinion pitch radius	112 mm
Pinion normal pressure angle	20°
Rack piston length	1440 mm
Piston diameter	200 mm
Designed stroke of the rack	546.875 mm
Guide ring width	20 mm
Guide ring thickness	2.5 mm
Number of LRG rollers	16
Diameter of LRG rollers	10 mm
Length of LRG rollers	30 mm

The actuator housing, cylinder barrel, hydraulic pipelines, mounting seat, and LRG track table are fabricated from 45 steel, while the pinion and rack are made of 18CrNiMo7-6 alloy steel. The guide track is constructed from 18Cr2Ni4WA alloy steel, and the LRG rollers and pinion shaft bearings are made of GCr15 alloy steel, which can achieve a surface hardness of approximately 58 HRC or higher under standard heat treatment conditions. The guide rings are manufactured from polytetrafluoroethylene (PTFE). The primary material properties are summarized in Table 2.

**Table 2:** Material properties.

Materials	Modulus of elasticity (MPa)	Density (kg/m^3^)	Poisson’s ratio
45 steel	2.06×10^5^	7850	0.27
18CrNiMo7-6	2.10×10^5^	7870	0.3
18Cr2Ni4WA	2.02×10^5^	7910	0.273
GCr15	2.08×10^5^	7800	0.3
PTFE	500	2170	0.46

**Fig. 3:**
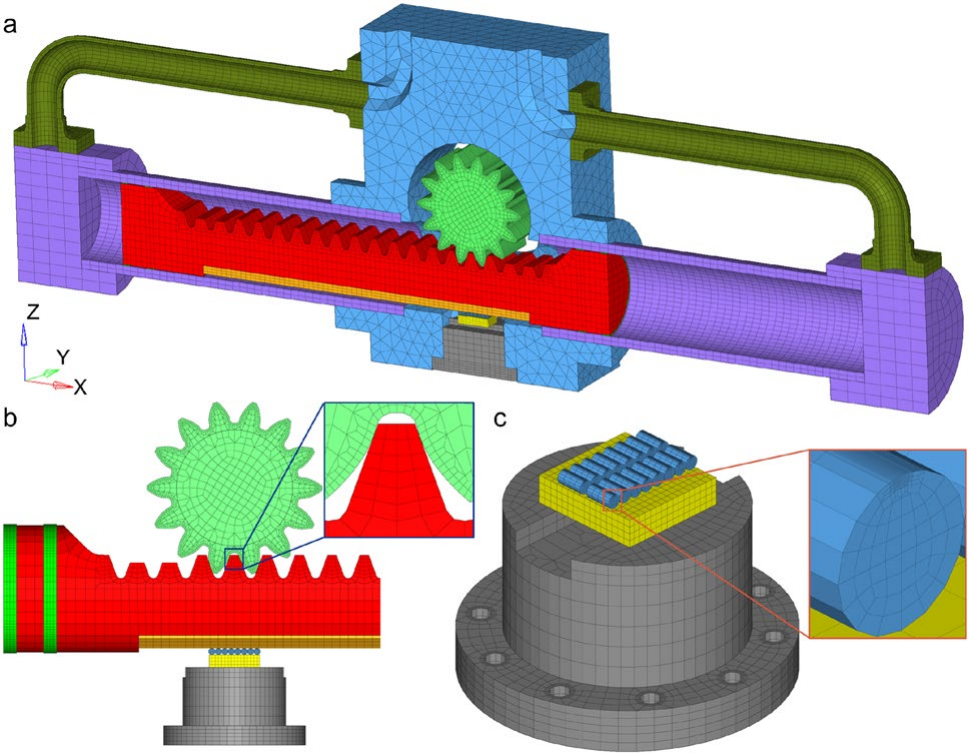
Finite element model of rack and pinion hydraulic actuator and support device. (**a**) Overall grid profile. (**b**) Rack and pinion pair. (**c**) LRG support device.

The actuator housing is discretized using tetrahedral elements (C3D4), while all other components are modeled with hexahedral elements (C3D8R). The overall finite element model of the rack and pinion hydraulic actuator and the LRG support device is shown in Fig. 3a. Local mesh refinement is applied in the pinion rack meshing region, the contact interface between guide rings and cylinder barrel, and the contact region between the LRG rollers and the guide track, to ensure accurate computation of contact interactions and local stress distributions, as illustrated in Fig. 3b and Fig. 3c.

**Fig. 4:**
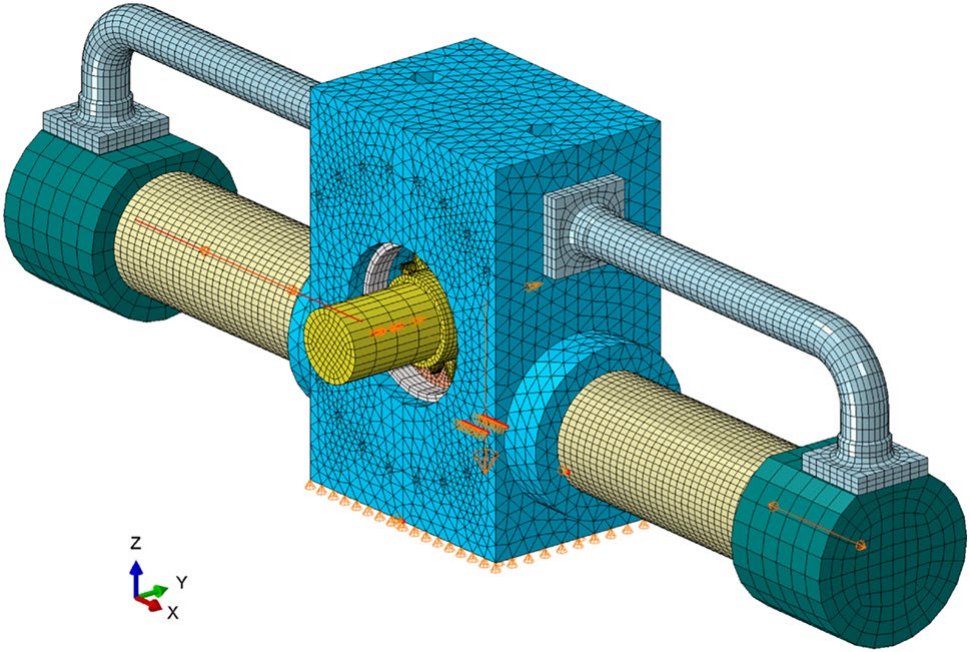
Schematic diagram of boundary conditions for the finite element model.

The established finite element model was imported into Abaqus/CAE to define the contact interactions and boundary constraints between components, as illustrated in Fig. 4. Tie constraints were applied between the rack and the inner surfaces of the guide rings, between the rack and the guide track, between the rollers and the track table, and between the track table and the mounting seat. Contact interactions are defined between the pinion and rack to simulate meshing process, while the outer surfaces of the guide rings and the inner wall of cylinder barrel, as well as the bearings and housing, are set as contact interfaces to transmit loads. An axial translational joint is assigned between the rack end and the cylinder face to apply hydraulic pressure.

Considering that friction at the rack pinion contact interface and the guide rings affects the load distribution of the support device, non-penetrating hard contact conditions were defined for these interfaces, incorporating both normal and tangential contact behaviors. The normal constraint was enforced using the augmented Lagrange method, while tangential friction was modeled using a penalty formulation with a friction coefficient of 0.1^23^. A similar contact definition was applied to the interface between the guide rings and the cylinder barrel, with the friction coefficient set to 0.05^24^. Additionally, given that the LRG rollers operate under rolling contact with the guide track and that adequate lubrication is maintained within the rack and pinion hydraulic actuator during operation, the frictional effect at this interface was neglected in the finite element analysis.

Additionally, the pinion shaft is coupled to the bearings through bushing elements, with radial stiffness assigned in both the longitudinal (X) and transverse (Y) directions as $k_{radial}$= 4.3×10^6^ N/mm. The expression for the radial stiffness based on Hertz contact theory is as follows:

1 $$k_{radial} = \frac{{F_{radial} }}{\delta }$$

2 $$F_{radial} = \frac{1}{2}\pi bp_{0} L$$

3 $$\delta = 2(\delta_{inner} + \delta_{outer} )$$

where $F_{radial}$ denotes the radial load on the bearing, $L$ is the contact length, $p_{0}$ represents the peak load density, $b$ is the half-width of the load distribution, which coincides with the contact half-width under Hertz elastic contact conditions. $\delta$ denotes the total deformation between the inner and outer bearing rings. In Hertz contact theory, the deformation of the inner and outer rings can be determined from the normal compression $\varepsilon$, as expressed in the following equations:

4 $$\varepsilon = \frac{{b^{2} }}{{R^{*} }}$$

5 $$b = \sqrt {\frac{{4FR^{*} }}{{\pi LE^{*} }}}$$

6 $$\frac{1}{{R^{*} }} = \frac{1}{{R_{1} }} + \frac{1}{{R_{2} }}$$

7 $$\frac{1}{{E^{*} }} = \frac{{1 - \mu_{1}^{2} }}{{E_{1} }} + \frac{{1 - \mu_{2}^{2} }}{{E_{2} }}$$

where $b$ is the Hertz contact half-width, $R^{*}$ denotes the equivalent radius of curvature, $F$ represents the normal load, $E^{*}$ is the equivalent elastic modulus, $R_{1}$ and $R_{2}$ are the radii of the two contacting bodies, respectively, $E$ and $\mu$ denote the elastic modulus and Poisson’s ratio of the contacting materials, respectively.

A composite kinematic pair is defined between the actuator housing and the pinion shaft to constrain their relative distance, while allowing a remote interaction torque about the Y-axis to be applied as the driving boundary condition for the pinion shaft. In addition, a fixed constraint is imposed at the base of the actuator housing.

**Fig. 5:**
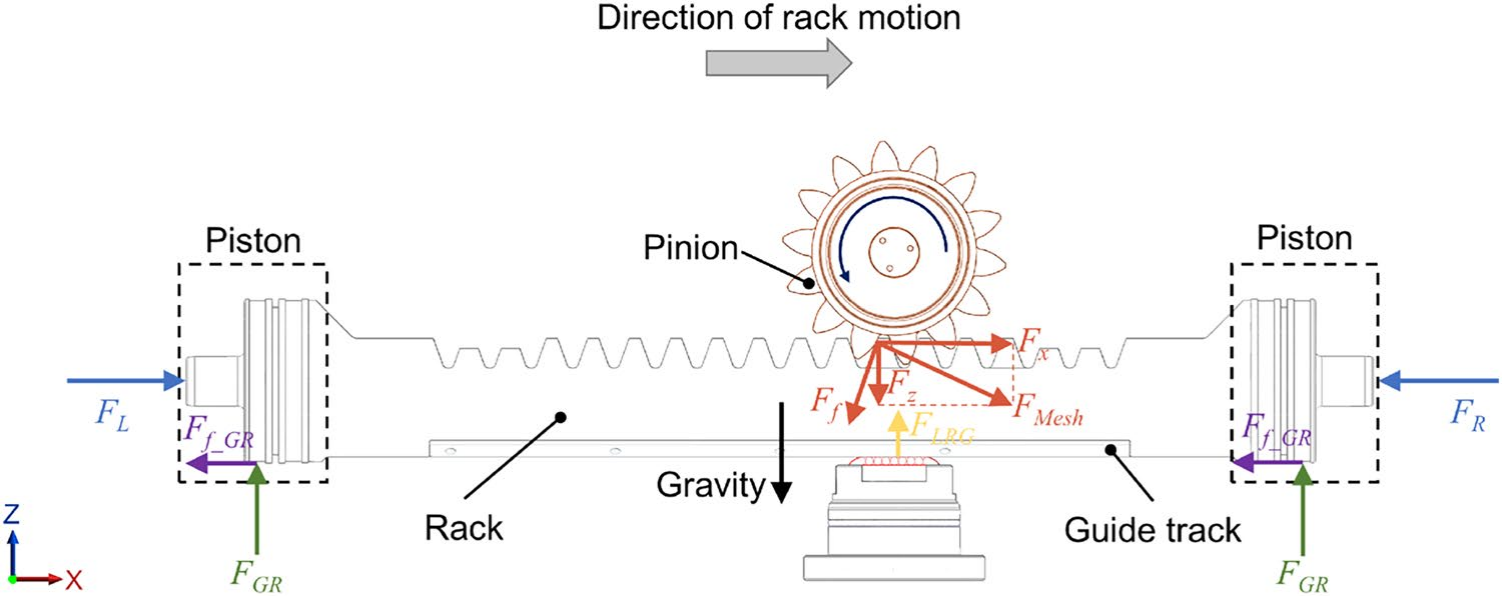
Free-body diagram of rack.

The free-body diagram of rack is shown in Fig. 5. Field measurements indicate that the maximum pressures in the left and right piston chambers of the rack and pinion hydraulic actuator are 30.43 MPa and 19.64 MPa, respectively. These piston chamber pressures are converted into equivalent constant loads and applied to both ends of the rack, as expressed by the following equations:

8 $$F_{L} = \frac{1}{4}\pi d_{piston}^{2} P_{L}$$

9 $$F_{R} = \frac{1}{4}\pi d_{piston}^{2} P_{R}$$

where $P_{L}$ and $P_{R}$ denote the maximum pressures in the left and right piston chambers of the actuator, $d_{piston}$ is the diameter of the rack piston. Based on Eq. (8) and Eq. (9), the constant loads acting on the left and right ends of the rack are calculated as 955.99 kN and 617.01 kN, respectively.

In addition, the designed stroke of the rack is 546.875 mm, and the pitch radius of the pinion is 112 mm. The corresponding angular displacement of the pinion shaft is 4.88 rad, as calculated via Eq. (10).

10 $$\theta = \frac{s}{r}$$

where $s$ is the designed stroke of the rack, $r$ is the pitch radius of the pinion. Accordingly, the rotational boundary condition of the pinion shaft is defined as a linear rotational displacement ranging from 0 to 4.88 rad.

### Mesh convergence verification of the finite element model

**Fig. 6:**
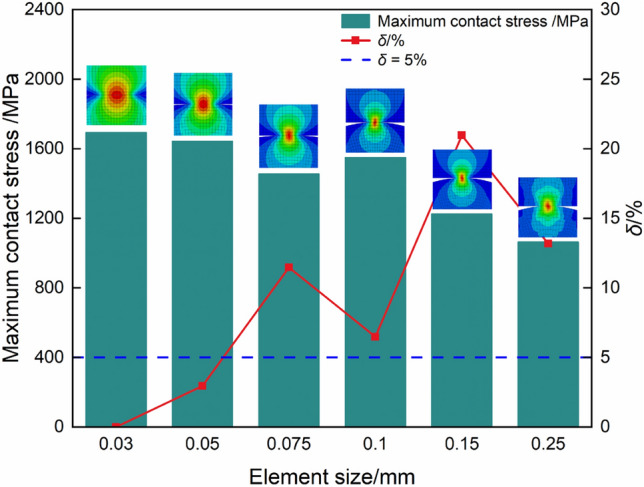
Results of the mesh convergence analysis.

To balance accuracy and computational efficiency in FEA, it is imperative to implement an appropriate local mesh refinement strategy in the contact region. In this study, a test model is established to investigate the convergence behavior with respect to the contact mesh size. Under identical boundary conditions, numerical simulations are performed using different mesh sizes in the contact region: 0.03, 0.05, 0.075, 0.10, 0.15, and 0.25 mm. The resulting contact stresses and corresponding computational times for each mesh configuration are extracted and comparatively analyzed, as summarized in Table 3 and illustrated in Fig. 6. The relative variation rate of the maximum contact stress between two successive mesh levels, denoted as $\delta$, is adopted as the convergence criterion^25^, defined as follows:

11 $$\delta = \frac{{\left| {S_{c}^{i + 1} - S_{c}^{i} } \right|}}{{S_{c}^{i} }} \times 100\%$$

where $S_{c}^{i + 1}$ and $S_{c}^{i}$ represent the maximum contact stresses corresponding to the size of the adjacent two-level mesh. In this study, mesh convergence is assumed to be achieved when $\delta < 5\%$, beyond which further mesh refinement is considered unnecessary.

**Table 3:** Calculation time and maximum contact stress for different mesh sizes.

Element size/mm	Maximum contact stress/MPa	*δ*/%	Computing time/s
0.03	1693.67	-	4210
0.05	1643.75	2.95	2664
0.075	1455.16	11.47	1959
0.10	1549.40	6.48	1032
0.15	1224.63	20.96	847
0.25	1063.34	13.17	558

As shown in Fig. 6, decreasing the mesh size in the contact region yields a progressively smoother contact stress distribution, with fewer abrupt variations in the transition zones. The morphology of the contact patch gradually approaches an approximately elliptical shape, which is consistent with Hertz contact theory^26^. Meanwhile, the maximum contact stress varies with the contact mesh size. When the mesh is refined to 0.05 mm, further refinement has a negligible effect on the maximum contact stress, with the relative variation rate $\delta < 5\%$, indicating that the numerical results have essentially converged at this mesh scale. Compared with the mesh size of 0.03 mm, the computational time at this scale is reduced by 36.7%, thereby significantly improving computational efficiency while maintaining adequate accuracy.

Based on the calculations from Eq. (5), the theoretical Hertz contact half-width for the LRG rollers is 0.15 mm. Combined with the above mesh convergence analysis, it can be concluded that satisfactory computational accuracy is achieved when the mesh size in the contact region is smaller than one-third of the contact half-width. This finding is consistent with the mesh discretization criteria for contact problems established in references^25^ and^27^. Accordingly, in all subsequent analyses, the mesh size in the contact region is specified to be no greater than one-third of the Hertz contact half-width. The final finite element model of the rack and pinion hydraulic actuator and LRG support device comprises a total of 99,684 elements and 102,928 nodes.

According to Hertz contact theory, the equations for calculating the maximum contact stress $p_{0}$ and the distribution of contact stress $p(x)$ along the X-axis are expressed as follows:

12 $$p_{0} = \frac{2F}{{\pi bL}}$$

13 $$p(x) = p_{0} \sqrt {1 - {{(x} \mathord{\left/ {\vphantom {{(x} b}} \right. \kern-0pt} b})^{2} }$$

**Fig. 7:**
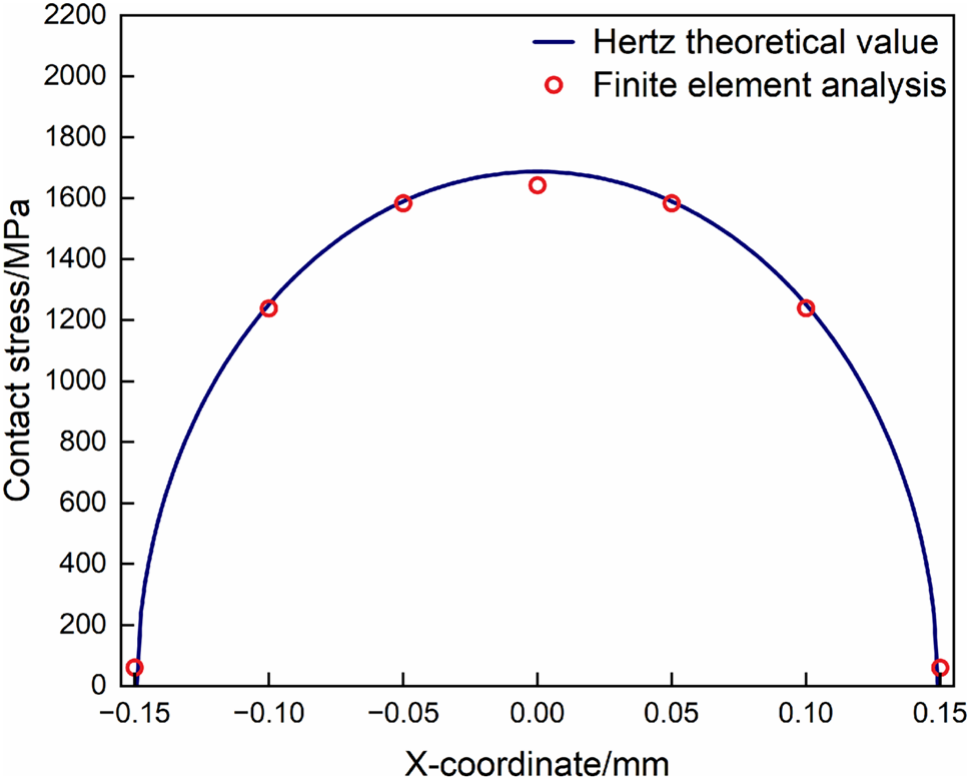
Distribution of contact stress.

The contact stress distributions along the X-axis, determined via Eq. (13) and FEA, are depicted in Fig. 7. The results indicate that both the magnitude and distribution of the contact stresses obtained by the FEA are in close agreement with the Hertz theoretical value, thereby further confirming the rationality of the selected local mesh refinement in the contact region and the reliability of the numerical results.

### Failure mechanism analysis based on finite element analysis

FEA of the heavy-duty rack and pinion hydraulic actuator and LRG support device was conducted using Abaqus/CAE. The simulation comprised two analysis steps. In the initial step, the loading condition was applied while the model remained stationary, with the applied load increasing linearly from zero to the prescribed value. In the subsequent step, the load was held constant while the actuator executed the full working stroke, during which the rack displacement was linearly increased from zero to 100% of the stroke.

**Fig. 8:**
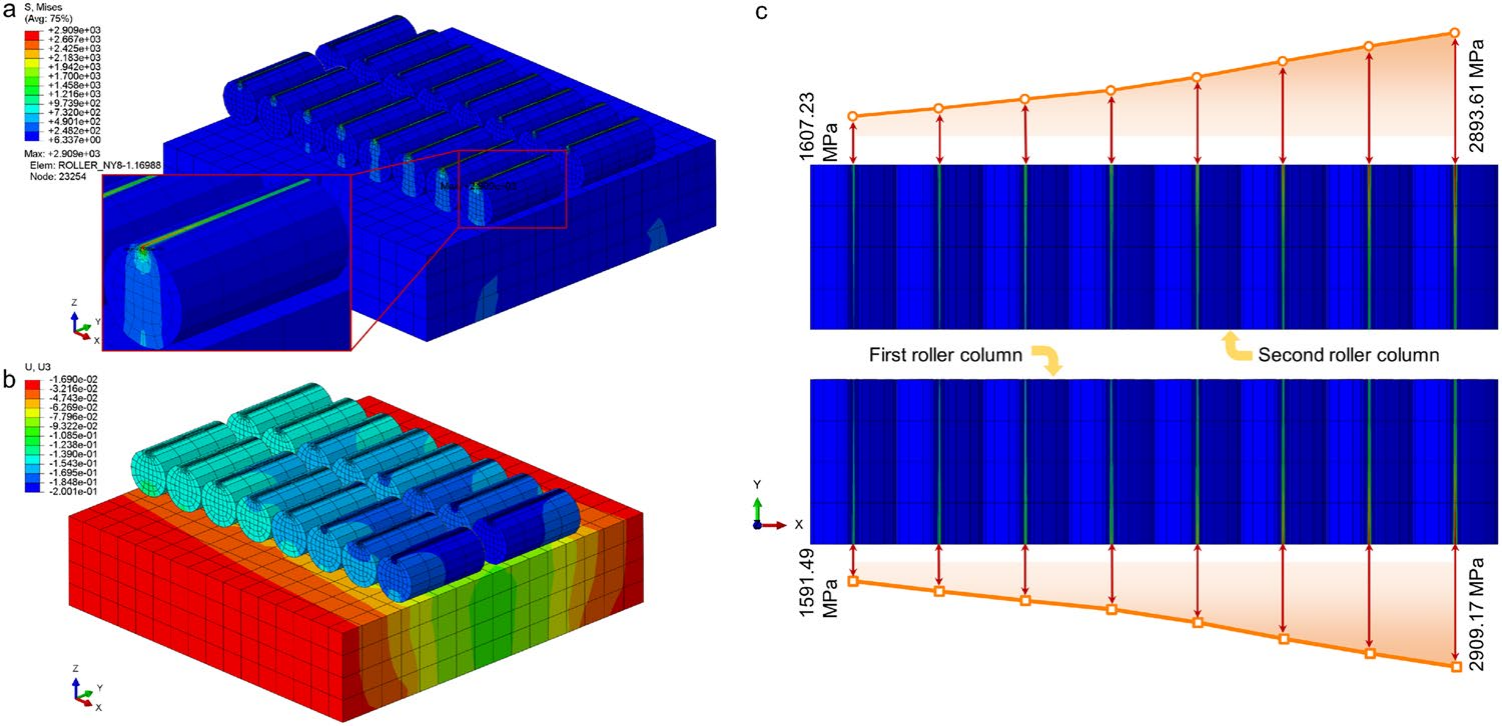
FEA results of the LRG support device. (**a**) Von Mises stress contour plot. (**b**) Vertical displacement contour plot. (**c**) Von Mises stress distribution diagram.

In this study, a linear elastic material model was employed, while failure assessment was performed based on the maximum Von Mises stress criterion by comparing the calculated stress with the allowable stress of the material. The allowable stress is defined as $[\sigma ] = {{\sigma_{s} } \mathord{\left/ {\vphantom {{\sigma_{s} } {n_{s} }}} \right. \kern-0pt} {n_{s} }}$, where $\sigma_{s}$ is the yield strength of the material, $n_{s}$ is the safety factor, which is set to 1.1 in this paper^28^. The LRG rollers are manufactured from GCr15 alloy steel, whose yield strength is 1786.8 MPa^29^, then the allowable stress of GCr15 is 1624.4 MPa. The von Mises stress contour plot of the overall LRG rollers at 0% working stroke, corresponding to the initial position, is shown in Fig. 8a. Table 4 summarizes the maximum von Mises stresses of the LRG rollers at different strokes. The results indicate that the maximum von Mises stress reaches 2909.17 MPa, occurring at the top of the rightmost roller in the first row, within the contact region between the roller and the guide track, which corresponds to the primary load transfer zone. This value significantly exceeds the allowable stress of the GCr15 alloy steel, indicating a crushing failure of the LRG roller under this loading condition. Fig. 8b presents the vertical displacement contour of the overall LRG rollers at the initial stroke, with the maximum vertical displacement of −0.20 mm, also occurring at the contact region of the rightmost roller in the first column. The distribution of the maximum Von Mises stresses for each LRG roller during the initial stroke is further extracted and illustrated in Fig. 8c.

**Table 4:** Maximum Von Mises stress of LRG rollers.

Stroke (%)	First roller column	Second roller column
0	2909.17 MPa	2893.61 MPa
10	2823.91 MPa	2806.51 MPa
20	2752.83 MPa	2734.73 MPa
30	2687.93 MPa	2664.84 MPa
40	2625.84 MPa	2602.07 MPa
50	2585.54 MPa	2584.04 MPa
60	2512.44 MPa	2483.73 MPa
70	2435.74 MPa	2406.27 MPa
80	2322.53 MPa	2290.89 MPa
90	1977.87 MPa	1950.28 MPa
100	1658.39 MPa	1630.82 MPa

Integration of Fig. 8a and Fig. 8c reveals that the rollers exhibit significant load non-uniformity along both the X and Y axes. In the Y-axis direction, the equivalent stress of rollers near the outer side is noticeably higher than that of rollers on the inner side of the contact region. Along the X-axis, the equivalent stress of the two roller columns shows a marked increasing trend in the positive X direction. Taking the first roller column as an example, the maximum Von Mises stress increases from 1591.49 MPa at the leftmost roller to 2909.17 MPa at the rightmost roller. The stress distribution results indicate a significant stress concentration at the outermost rollers, thereby substantiating the analysis in Section "Failure description" regarding the significant local load non-uniformity of the LRG support device under actual operating conditions.

**Fig. 9:**
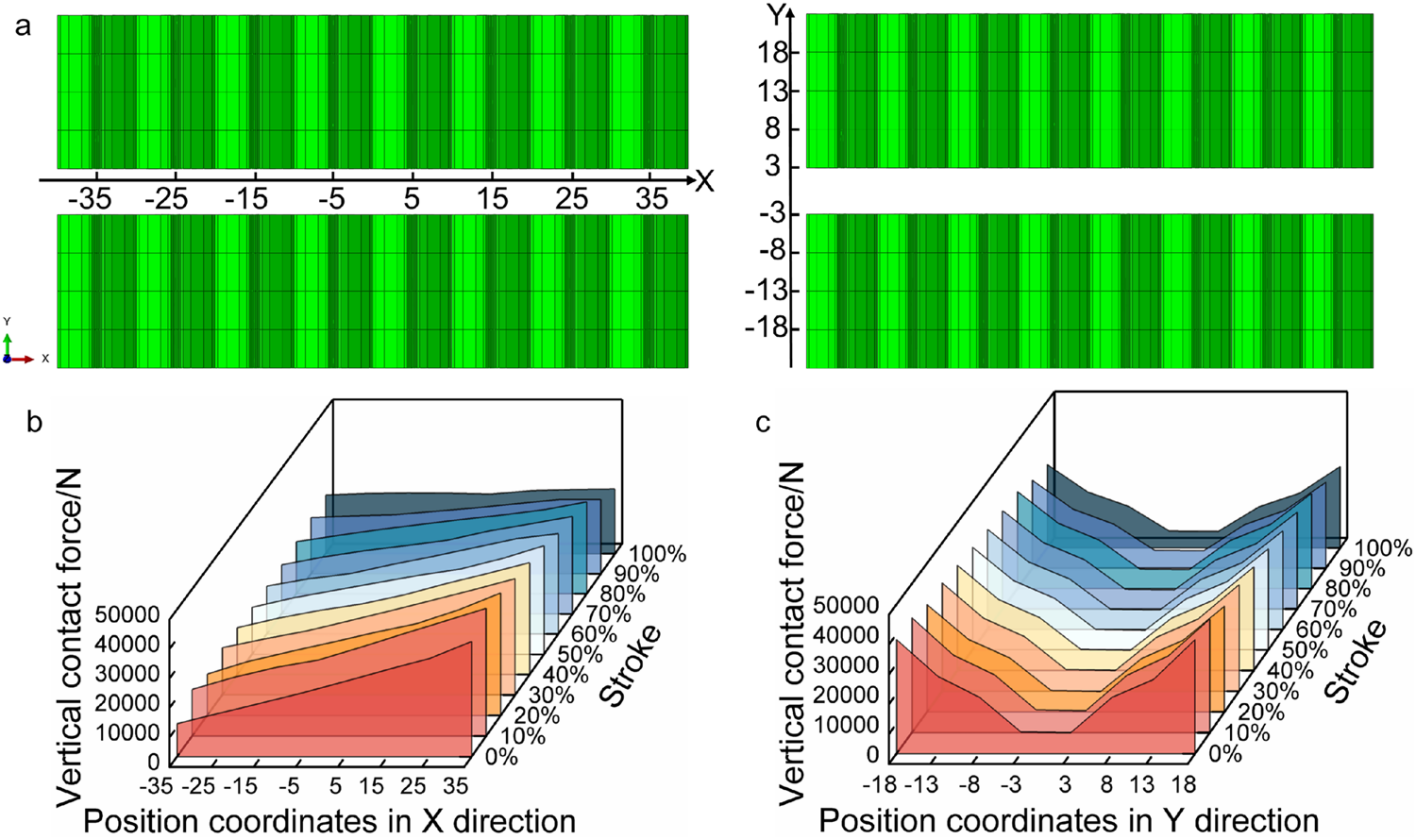
(**a**) Sampling position coordinate diagram. (**b**) Vertical contact force in X-direction. (**c**) Vertical contact force in Y-direction.

Since contact stress represents the result of contact forces distributed over a finite contact area, its distribution is highly dependent on the load transmission and support stiffness matching at the system level. Consequently, it is imperative to further analyze the load distribution patterns between different rollers from the perspective of contact forces to reveal the fundamental causes of load eccentricity and the associated failure mechanisms. To this end, sampling node paths were selected at the contact interface between the tops of the rollers and the rack guide track. Vertical nodal contact forces were extracted across the 0% to 100% operational stroke range according to the coordinates illustrated in Fig. 9a. The resulting load distributions along the X and Y axes are presented in Fig. 9b and Fig. 9c, respectively. Notably, the vertical contact force in the X-direction was derived by superimposing the contact forces from the two rows of rollers. In this study, the degree of load distribution non-uniformity in the supporting system is evaluated using the eccentric load factor, defined as follows:

14 $$\eta = \frac{{F_{\max } }}{{\overline{F}}}$$

where $F_{\max }$ is the maximum vertical load among the rollers, $\overline{F}$ is the average vertical load of the rollers.

**Fig. 10:**
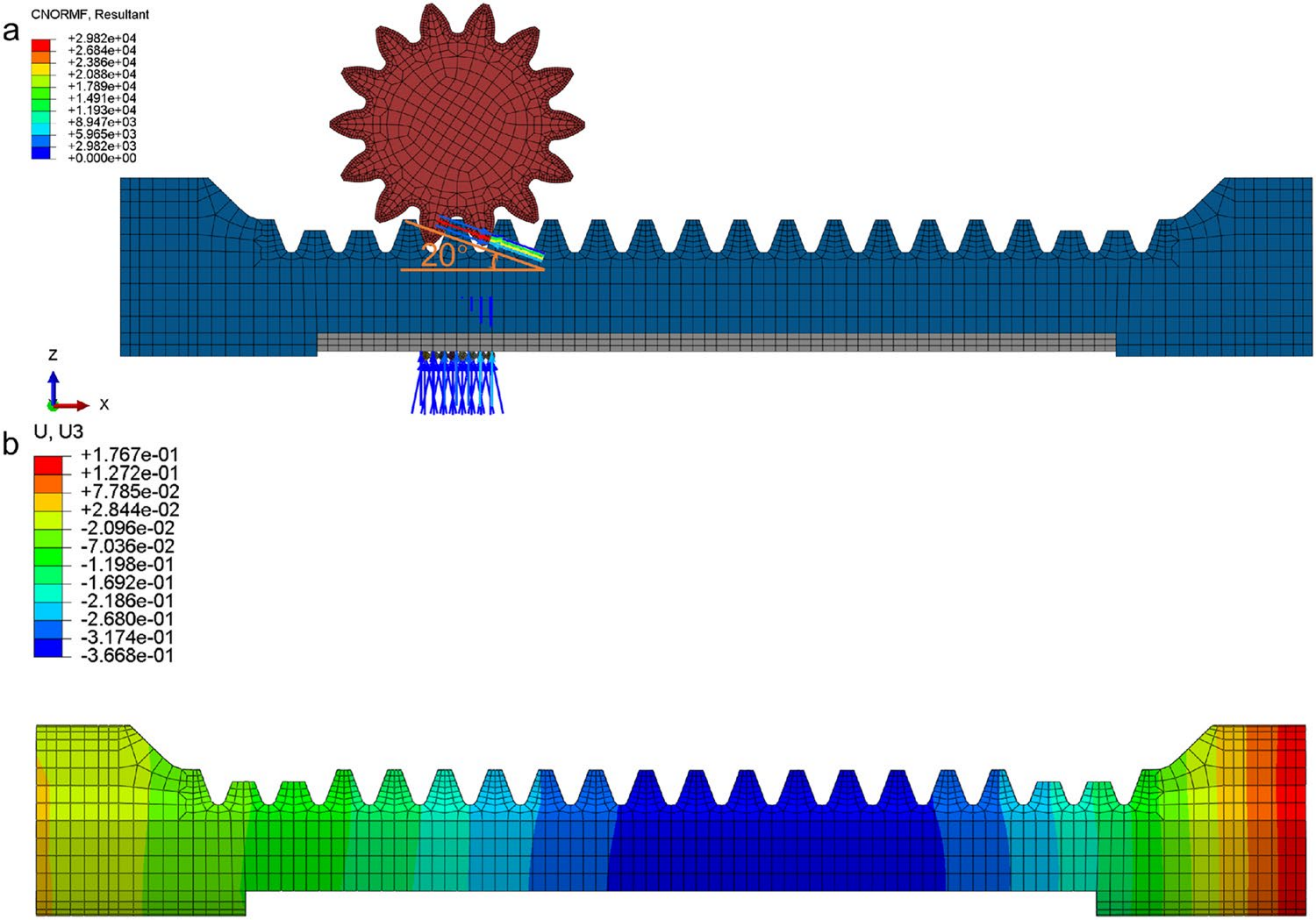
(**a**) Schematic diagram of normal contact forces in a pinion rack pair. (**b**) Contour plot of vertical deformation of the rack.

As illustrated in Fig. 9b, the LRG rollers exhibit significant eccentric loading along the X-axis throughout the entire rack stroke. This phenomenon is most severe at the initial stroke, where the maximum eccentric load factor reaches 1.68. In conjunction with the normal contact force vectors of the rack pinion pair illustrated in Fig. 10a, the involute meshing characteristics dictate an approximately 20° angle between the normal contact force and the direction of rack motion, thereby introducing a substantial vertical force component. Furthermore, the vertical displacement contour plot of the rack shown in Fig. 10b indicates a downward displacement of 0.37 mm at the center of rack, accompanied by upward displacements at both ends, confirming the occurrence of rack flexural deformation. The combined effects of the meshing force components and rack flexural deformation jointly result in the load eccentricity along the X-axis. In addition, the progressive increase in stress from the leftmost roller to the rightmost roller observed in Fig. 8c directly corresponds to the X-direction load eccentricity quantified in Fig. 9b. This eccentricity originates from the coupled effects of the vertical force component of the pinion, as shown in Fig. 10a, and the flexural deformation of the rack, as illustrated in Fig. 10b. Fig. 9c demonstrates that eccentric loading is also present along the Y-axis, with the vertical contact forces on the inner side of the rollers are consistently lower than those on the outer side. The eccentric load level exhibits only minor variation across different stroke positions, with a maximum eccentric load factor of 1.72. Correlating this with Fig. 8b, the Y-axis load eccentricity primarily results from the subsidence of the central region of the track table beneath the LRG rollers, causing the outer-side rollers to bear the majority of the rack load and thereby inducing eccentric loading along the Y-axis.

**Fig. 11:**
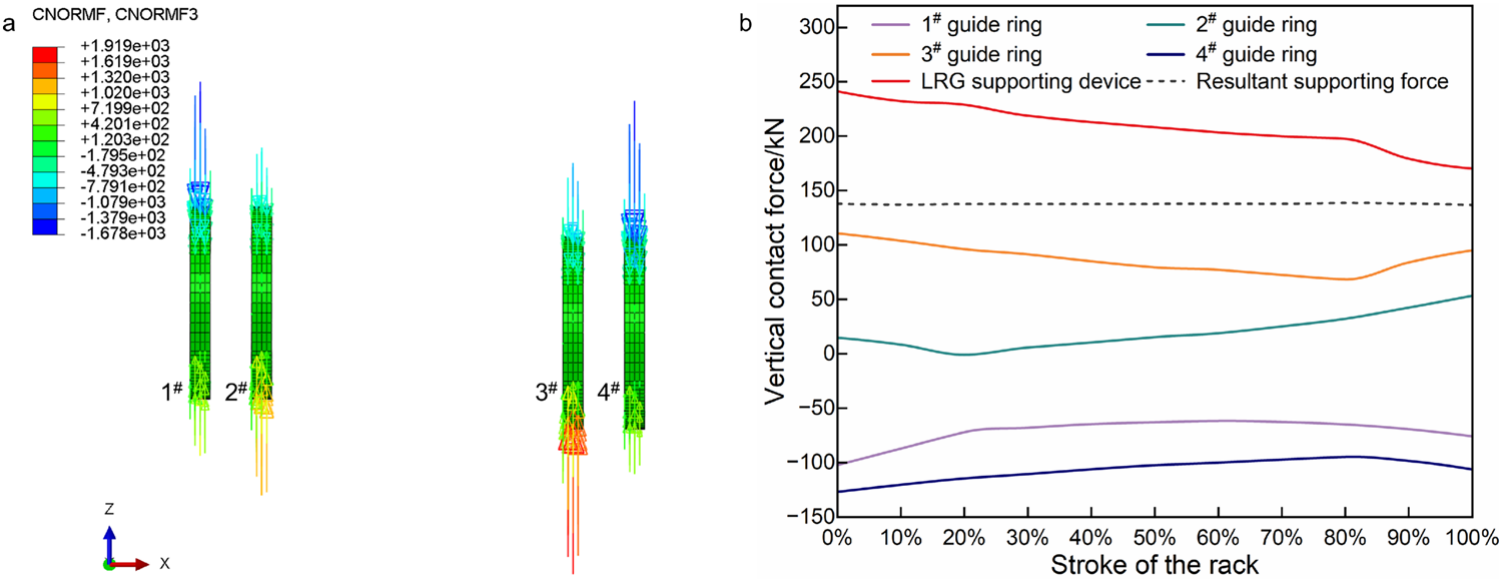
(**a**) Vertical contact force direction of the guide ring. (**b**) Comparison of vertical contact force.

To address the excessive wear of the guide rings on both sides of the rack, the vertical contact forces exerted by each guide ring on the rack were analyzed, as shown in Fig. 11. Fig. 11a indicates that the vertical support forces provided by guide rings 1^#^ and 4^#^ act primarily along the negative Z-axis direction, whereas the forces from guide rings 2^#^ and 3^#^ are generally oriented in the positive Z-axis direction. This distribution further confirms that the center of the rack undergoes downward vertical displacement and subsequent flexural deformation. Fig. 11b demonstrates that the contact force magnitudes for guide rings 3^#^ and 4^#^ are higher than those for 1^#^ and 2^#^, thereby further validating the presence of load eccentricity in the system. The LRG support device bears most of the vertical load, with the average supporting force of 208.44 kN, while the supporting forces of the guide rings range from −126.80 kN to 110.68 kN. These results indicate that, under heavy-duty operating conditions, the guide rings exhibit pronounced unintended load-bearing participation, reflecting an irrational load transfer path and non-uniform load distribution in LRG support device. Consequently, the guide rings are subjected to excessive vertical loads, leading to severe wear. Furthermore, Fig. 11b demonstrates that the resultant supporting force provided by the LRG support device and the guide rings remains substantially constant across different strokes, with an average value of 137.63 kN.

Based on the FEA results, it can be concluded that the roller crushing failure of support device in the heavy-duty rack and pinion hydraulic actuator originates from the simultaneous load eccentricity of the LRG rollers in both the X and Y directions. This combined eccentric loading causes the local contact stress at the end rollers to exceed the material yield limit, ultimately leading to elastic-plastic deformation of the end rollers.

## Design and validation of the improved support device

Based on the FEA results presented in Section "Finite element analysis and failure mechanism investigation", the single-sided roller crushing failure of the LRG support device is primarily attributable to the combined effects of local load non-uniformity, excessive local contact forces, and insufficient adaptability to rack flexural deformation. In view of the deficiencies identified in the LRG support device within this heavy-duty rack and pinion hydraulic actuator application, two alternative support schemes are proposed in this study, which are sliding support device and track roller support device.

### Sliding support device

The sliding support device employs surface contact between the slider and the rack guide track, replacing the discrete point/line contact inherent in the original LRG support device. By substantially expanding the effective contact area, this scheme reduces local contact stresses, thereby preventing the elastic-plastic transition and crushing failure of the LRG rollers, and is thus more suitable for heavy-duty operating conditions. Furthermore, the sliding interface permits a degree of relative displacement under heavy loads, which enhances the adaptability of the supporting system to rack flexural deformation and promotes a more uniform distribution of supporting reactions within the contact region.

**Fig. 12:**
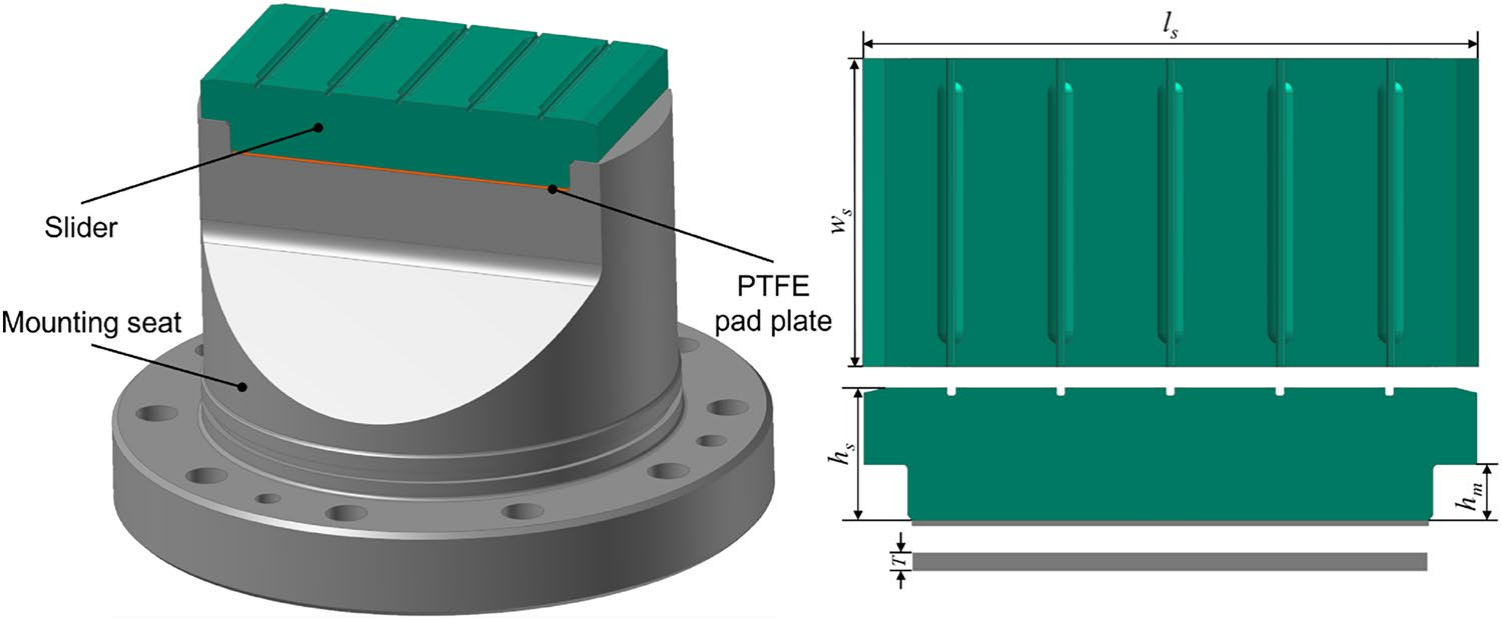
Geometric model of sliding support device.

The sliding support device primarily comprises a slider, a PTFE pad plate, and a mounting seat, with its geometric model illustrated in Fig. 12. The upper surface of the slider features lubrication grooves designed to improve the tribological and frictional characteristics of the contact interface. The slider is fabricated from a copper-aluminum-nickel alloy (CuAl10Ni), which exhibits excellent wear resistance and load-carrying capacity^30,31^, with a yield strength of 300 MPa^32^. The PTFE pad plate, characterized by a low elastic modulus and a certain degree of compressibility^33,34^, can effectively absorb local load peaks and thereby mitigate stress concentration within the contact region.

The planar geometric dimensions of the slider directly determine the load-carrying capacity of the contact interface. To ensure structural reliability, the design criterion requires that the operating contact pressure $p$ of the slider does not exceed the allowable contact pressure $[p]$ of the material, as defined by the following expression:

15 $$p = \frac{F}{{A_{c} }} \le [p]$$

16 $$A_{c} = l_{s} w_{s}$$

17 $$[p] = \alpha \left[ {\sigma_{c} } \right] = \alpha \frac{{\sigma_{s} }}{{n_{s} }}$$

where $A_{c}$ is the effective contact area; $l_{s}$ and $w_{s}$ are the length and width of the slider contact surface, respectively; $[p]$ is the allowable contact pressure of the material; $[\sigma_{c} ]$ is the allowable compressive stress; $\sigma_{s}$ is the material yield strength; $n_{s}$ and $\alpha$ are the safety factor and correction coefficient, respectively. In this study, based on engineering experience and relevant literature^28^, $n_{s}$ and $\alpha$ are assigned values of 1.5 and 0.3.

Based on the computational results in Section "Failure mechanism analysis based on finite element analysis", the rack exhibits significant flexural deformation. Consequently, the design of the slider dimensions prioritizes increasing the contact surface length $l_{s}$. Defining the aspect ratio of the slider contact surface as $\lambda = 2$, the expressions for the slider length and width are as follows:

18 $$l_{s} = \lambda w_{s}$$

19 $$w_{s} = \sqrt {\frac{{n_{s} F}}{{\alpha \lambda \sigma_{s} }}}$$

The thickness of the slider determines its structural stiffness and bending strength. To conservatively evaluate the bending strength, the slider is approximated as a rectangular-section beam subjected to a uniformly distributed load, with the bending stress calculated as follows:

20 $$\sigma_{b} = \frac{{3Fw_{s} }}{{4l_{s} h_{s}^{2} }} \le [\sigma_{b} ]$$

where $h_{s}$ is the slider thickness, $[\sigma_{b} ]$ is the allowable bending stress. The expression for the slider thickness can be derived as follows:

21 $$h_{s} = \sqrt {\frac{3F}{{4\lambda [\sigma_{b} ]}}}$$

Based on the above equations, the structural dimensions of the sliding support device were calculated and subsequently rounded for engineering feasibility. Considering the spatial constraints of the rack and pinion hydraulic actuator and the assembly requirements of the mounting seat, the slider dimensions were constrained to $l_{s} \le 152\;{\mathrm{mm}}$, $w_{s} \le 80\;{\mathrm{mm}}$, and $h_{s} \le 28\;{\mathrm{mm}}$. The key dimensional parameters of the slider shown in Fig. 12 are listed in Table 5.

**Table 5:** Key structural dimension parameters of the sliding support device.

Parameters	Symbol	Value
Slider length	$l_{s}$	140 mm
Slider width	$w_{s}$	70 mm
Slider thickness	$h_{s}$	25 mm
Slide mounting height	$h_{m}$	10.5 mm
PTFE pad thickness	$T$	1 mm

### Track roller support device

**Fig. 13:**
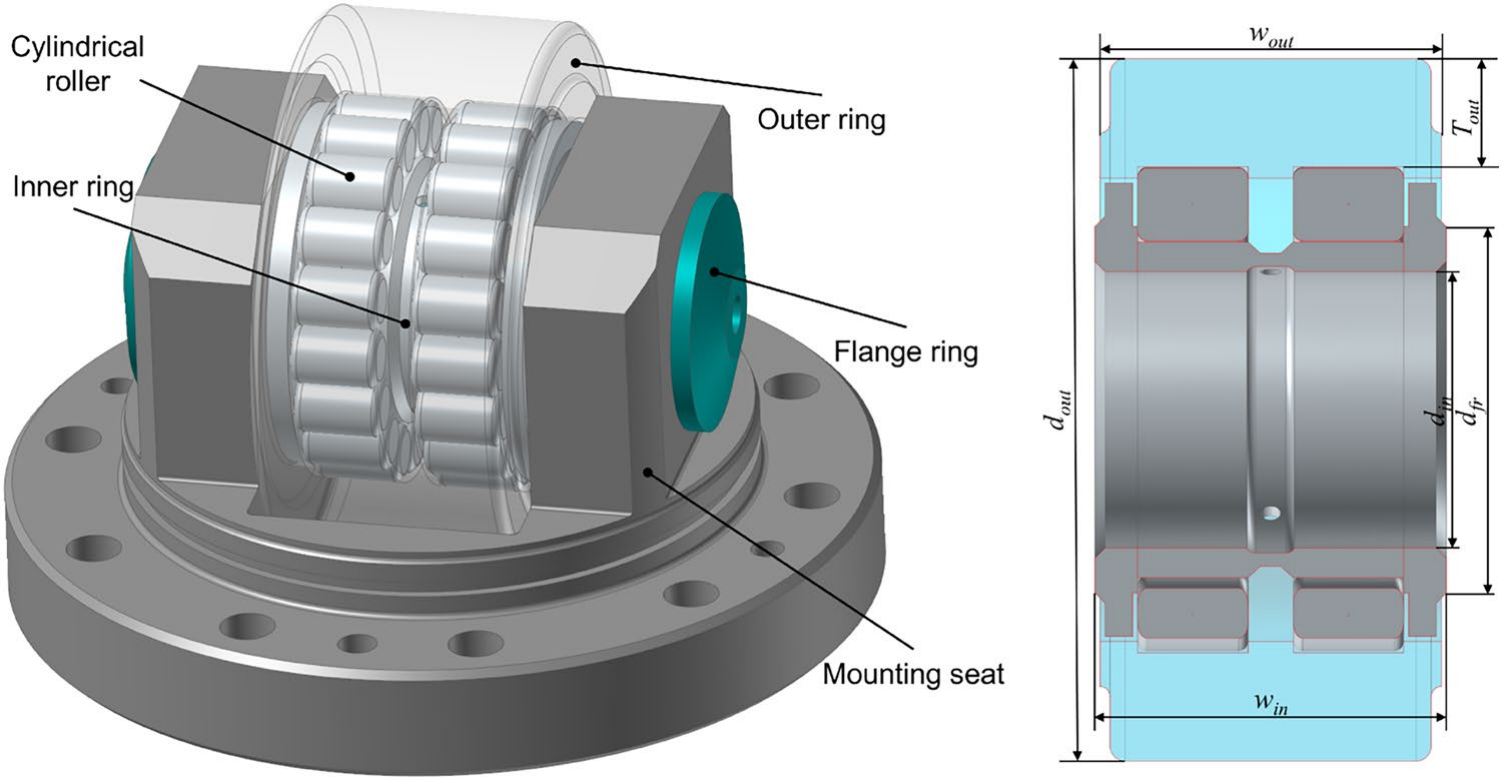
Geometric model of track roller support device.

The track roller support device replaces the original LRG support with rolling bearing units that provide higher radial load capacity, thereby enhancing load-carrying performance and operational reliability under heavy loads. This device primarily consists of an outer ring, an inner ring, cylindrical rollers, flange rings, a pin shaft, and a mounting seat, as illustrated in Fig. 13.

The structural architecture of the track roller support device is based on double-row cylindrical roller bearings featuring an inner ring. The outer ring features a thick-walled design that effectively withstands substantial axial loads induced by misalignment or tilting, while mitigating edge contact and improving load distribution. The outer ring of the track roller is made of GCr15 alloy, whereas the mounting seat, flange ring and pin shaft are fabricated from 45 steel.

The outer ring of the track roller is in line contact with the rack guide track, and the cylindrical rollers are also in line contact with the inner and outer rings. Assuming that the external load is primarily shared by the two rows of cylindrical rollers, the contact stress between the rollers and the inner raceway can be estimated as follows, based on Eq. (5) and Eq. (12):

22 $$\sigma_{H} = \sqrt {\frac{{Q_{\max } E^{*} }}{{\pi l_{r} R^{*} }}} \le [\sigma_{H} ]$$

where $Q_{\max } = {{KF} \mathord{\left/ {\vphantom {{KF} Z}} \right. \kern-0pt} Z}$ is the maximum load per roller, $F$ is the total external load, $K$ is the load distribution factor, $Z$ is the number of rollers per row, $l_{r}$ is the length of the rollers. In practical operation, the external load acting on the track roller is not uniformly distributed among all rollers. Only the rollers located in the upper load-carrying region primarily support the load. The load distribution factor $K$ is introduced to account for this non-uniform load sharing, with a value set at 4.0^35^.

The equivalent elastic modulus $E^{*}$ and the equivalent radius of curvature $R^{*}$ are given by the following equations:

23 $$E^{*} = \frac{E}{{2(1 - \mu^{2} )}}$$

24 $$R^{*} = \frac{{d_{r} d_{ir} }}{{2(d_{r} + d_{ir} )}}$$

where $d_{r}$ is the roller diameter, $d_{ir}$ is the inner ring outer diameter with $d_{ir} = d_{m} - d_{r} = (k_{m} - 1)d_{r}$, $d_{m}$ and $k_{m}$ denote the pitch circle diameter and the pitch circle factor, respectively.

Given the length to diameter ratio of the cylindrical roller as $\lambda_{r} = 1.6$, the analytical expression for the minimum roller diameter can be derived from Eqs. (22) to (24):

25 $$d_{r} = \sqrt {\frac{{KFEk_{m} }}{{\pi (k_{m} - 1)Z\lambda_{r} [\sigma_{H} ]^{2} (1 - \mu^{2} )}}}$$

Based on Eq. (25), the outer ring width $w_{out}$, outer ring inner diameter $d_{or}$, outer ring outer diameter $d_{out}$, and inner ring inner diameter $d_{in}$ can be calculated using the following equations:

26 $$w_{out} = 2(\lambda_{r} d_{r} + \delta ) + w_{mid}$$

27 $$d_{or} = d_{m} + d_{r} = (k_{m} + 1)d_{r}$$

28 $$d_{out} = d_{or} + 2T_{out}$$

29 $$d_{in} = d_{ir} - 2T_{in}$$

where $\delta$ is the distance from the end of the roller to the end face of the outer ring, $w_{mid}$ is the width of the central rib, $T_{out}$ and $T_{in}$ are the outer ring and inner ring thickness, respectively.

The outer ring of the heavy-duty track roller can be equivalently modeled as a thick-walled circular ring under radial load, and the maximum bending stress is given as:

30 $$\sigma_{b} = \frac{{3Fd_{out} }}{{4\pi T_{out}^{2} }} \le [\sigma_{b} ]$$

Based on Eq. (30), the minimum outer ring thickness can be determined using the following expression:

31 $$T_{out} = \sqrt {\frac{{3Fd_{out} }}{{4\pi [\sigma_{b} ]}}}$$

Utilizing the above formulations, the structural dimensions of the track roller support device were calculated and subsequently rounded for engineering feasibility. It should be noted that these equations provide a conservative estimation specifically for heavy-duty track rollers rather than standard rolling bearings. Taking into account the spatial constraints of the rack and pinion hydraulic actuator and the assembly requirements of the mounting seat, the principal structural dimensions of the track roller support assembly were finally determined. The key dimensional parameters of the track roller shown in Fig. 13 are listed in Table 6.

**Table 6:** Key structural dimension parameters of the track roller support device.

Parameters	Symbol	Value
Outer ring width	$w_{out}$	68 mm
Inner ring width	$w_{in}$	70 mm
Outer ring thickness	$T_{out}$	21.5 mm
Outer ring outer diameter	$d_{out}$	140 mm
Inner ring inner diameter	$d_{in}$	55 mm
Outside diameter flange ring	$d_{fr}$	73 mm
Cylindrical roller diameter	$d_{r}$	15 mm
Cylindrical roller length	$l_{r}$	24 mm

### Validation of the effectiveness of the improved support device

**Fig. 14:**
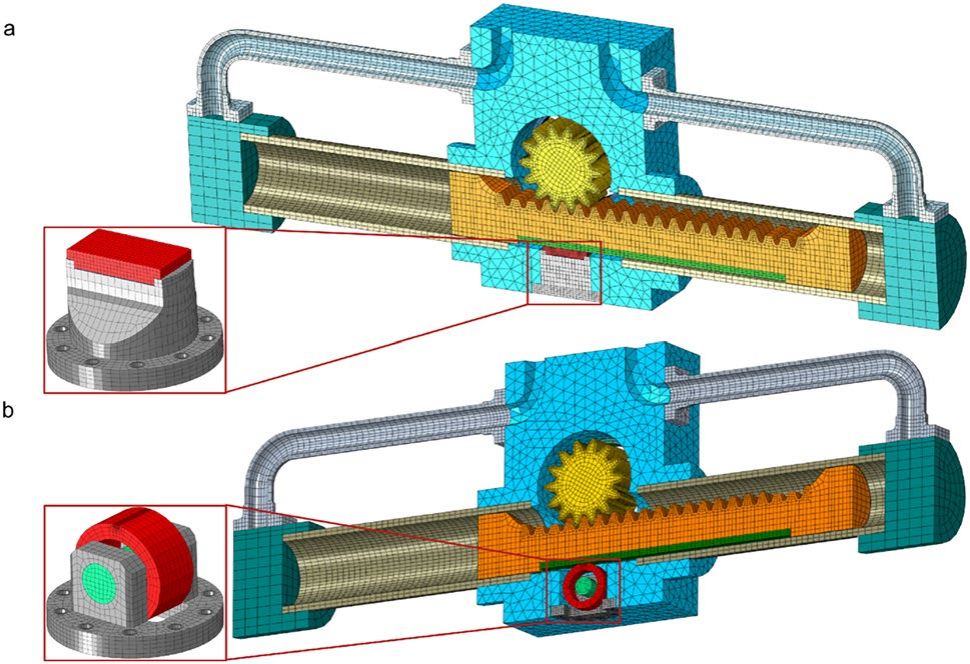
Finite element models of the improved schemes. (**a**) Sliding support device. (**b**) Track roller support device.

To verify the effectiveness of the sliding support and track roller support devices, finite element models of both improved support devices were established, as shown in Fig. 14. Both models were discretized using hexahedral elements (C3D8R). Since the load within the track roller support device is transmitted through the outer ring, cylindrical rollers, and inner ring, its mechanical response is strongly influenced by radial stiffness. Consequently, the outer ring and pin shaft are interconnected within the model via a bushing connection, where the bushing stiffness is employed to equivalently represent the radial stiffness between the inner and outer rings. Based on Eq. (1) through Eq. (3), the radial stiffness of the track roller is calculated to be 4×10^6^ N/mm. Additionally, the local mesh refinement in the contact region was determined according to the contact half-width between the outer ring and the rack guide track. Utilizing Eq. (5), the theoretical contact half-width was calculated as 1.52 mm, and the element size in the contact region was set to 0.4 mm.

In Abaqus/CAE, the two improved support devices were analyzed under the same analysis steps and loading conditions as those applied to the LRG support device. For the sliding support device, sliding contact occurs between the slider and the guide track. Accordingly, a non-penetrating hard contact condition was defined between the two surfaces, incorporating both normal and tangential contact behaviors. The normal constraint was implemented via the augmented Lagrange method, while tangential friction was described by a penalty formulation, in which the friction coefficient was set to 0.1^36^.

**Fig. 15:**
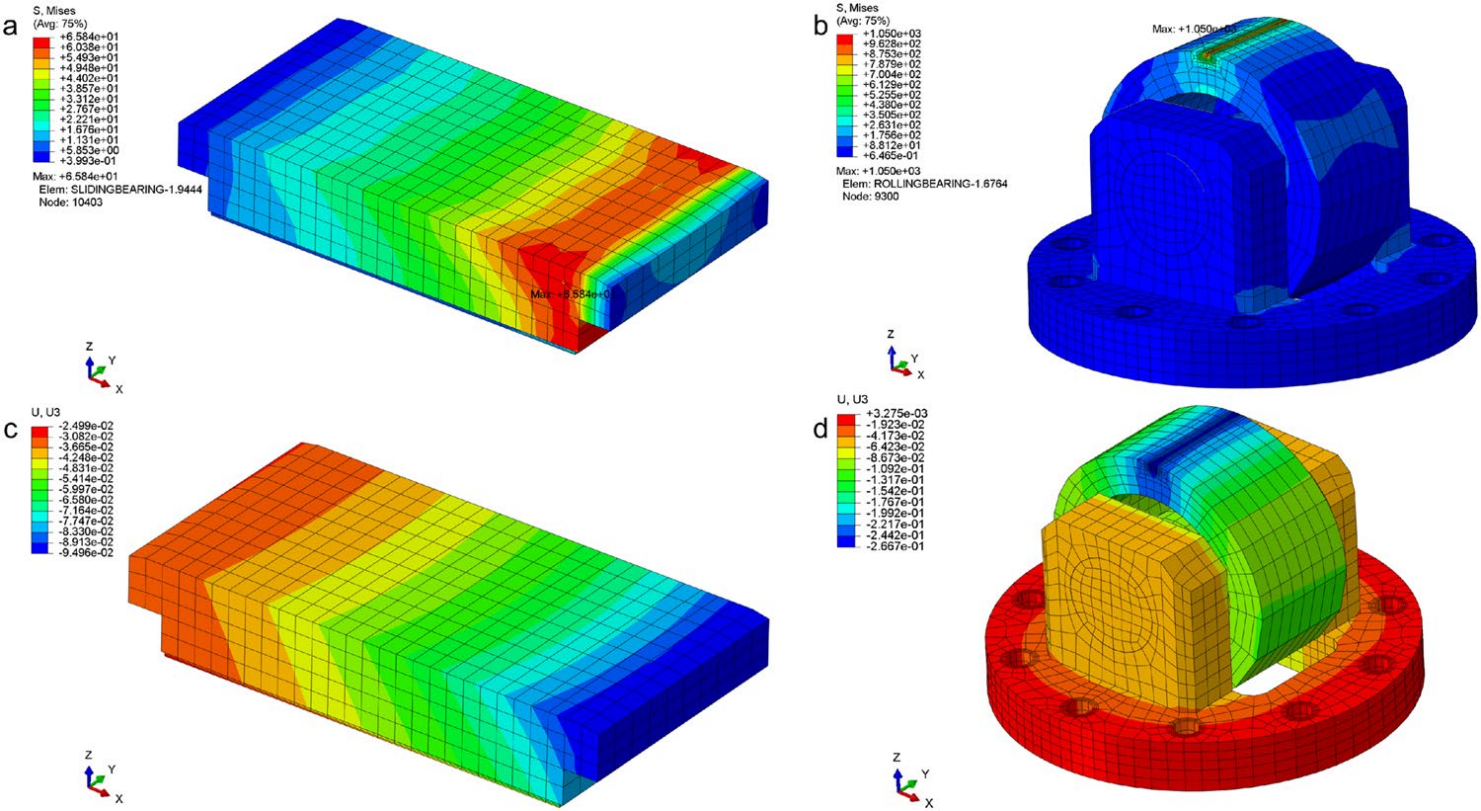
FEA results of the improved support devices. (**a**) Von Mises stress of slider. (**b**) Von Mises stress of track roller. (**c**) Vertical displacement of slider. (d) Vertical displacement of track roller.

The mechanical responses of the heavy-duty rack and pinion hydraulic actuator equipped with the improved supports were obtained via FEA. Fig. 15 presents the Von Mises stress and vertical displacement contour plots of the slider and the track roller, while Table 7 summarizes the maximum Von Mises stresses of the slider and roller at different stroke positions.

**Table 7:** Maximum Von Mises stresses of slider and track roller.

Stroke (%)	Slider	Track roller
0	65.83 MPa	1050.28 MPa
10	64.12 MPa	957.10 MPa
20	63.45 MPa	1025.19 MPa
30	61.07 MPa	1032.22 MPa
40	59.88 MPa	995.71 MPa
50	59.60 MPa	1014.54 MPa
60	59.55 MPa	1002.48 MPa
70	56.54 MPa	957.67 MPa
80	55.24 MPa	991.93 MPa
90	51.76 MPa	926.84 MPa
100	46.39 MPa	892.69 MPa

The FEA results indicate that the maximum vertical displacements for the slider and track roller are −0.095 mm and −0.267 mm, respectively. The maximum Von Mises stress of the slider reaches 65.83 MPa at the initial stroke, remaining well below the allowable stress of CuAl10Ni. The maximum Von Mises stress of the track roller is 1050.28 MPa, occurring at the initial stroke, and likewise does not exceed the allowable stress of GCr15. By combining the data presented in Tables 4 and 7, it’s evident that both improved schemes exhibit significantly lower maximum stress levels than the original LRG support device. The stress reduction in the sliding support device is primarily attributed to the transformation of point/line contact into surface contact, which substantially increases the effective contact area. In contrast, the track roller support device achieves stress reduction through improved load distribution characteristics and the higher radial load-carrying capacity of the bearing structure.

**Fig. 16:**
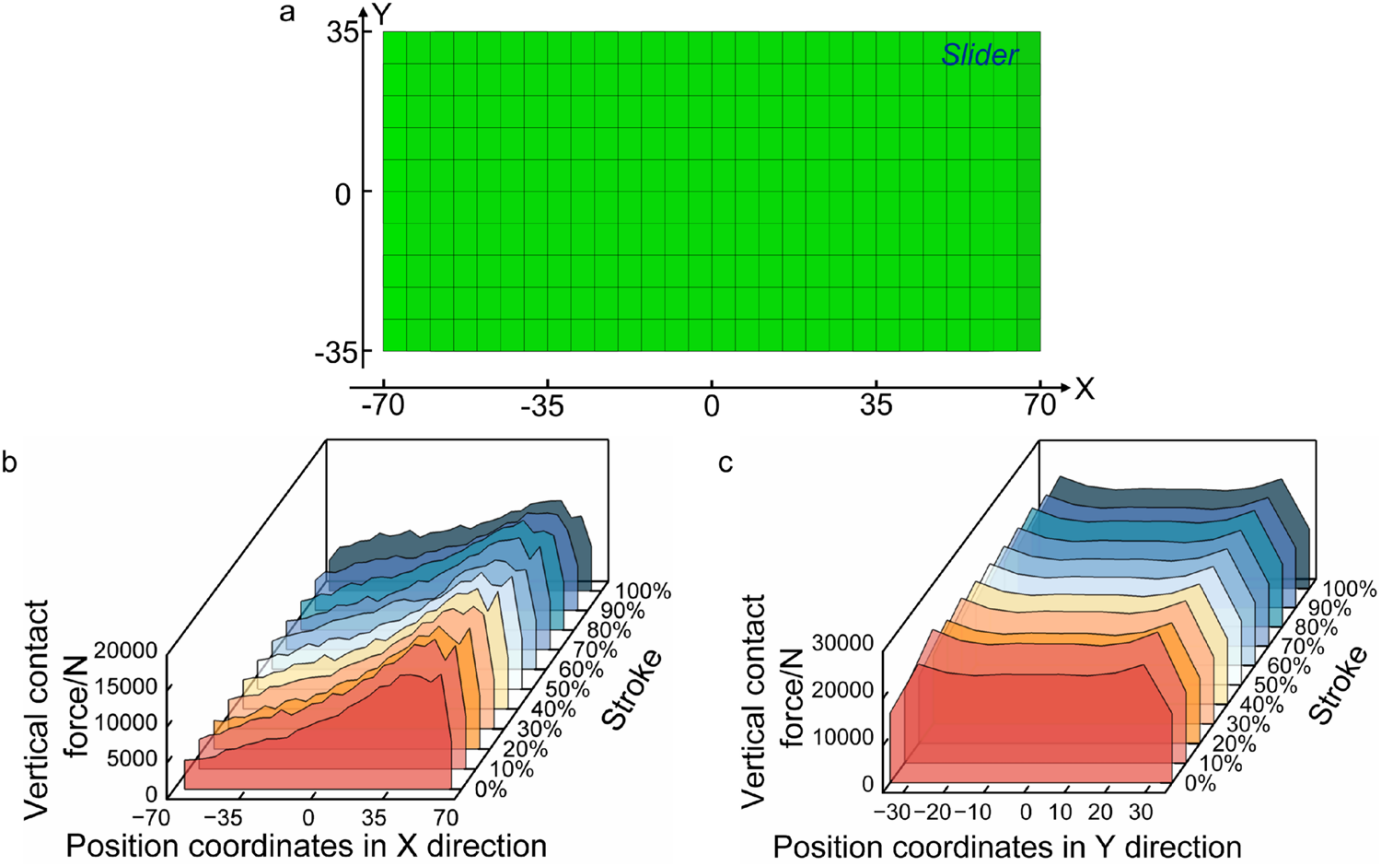
Vertical contact forces of the slider at different stroke. (**a**) Sampling position coordinate diagram. (**b**) Vertical contact force in the X direction. (**c**) Vertical contact force in the Y direction.

**Fig. 17:**
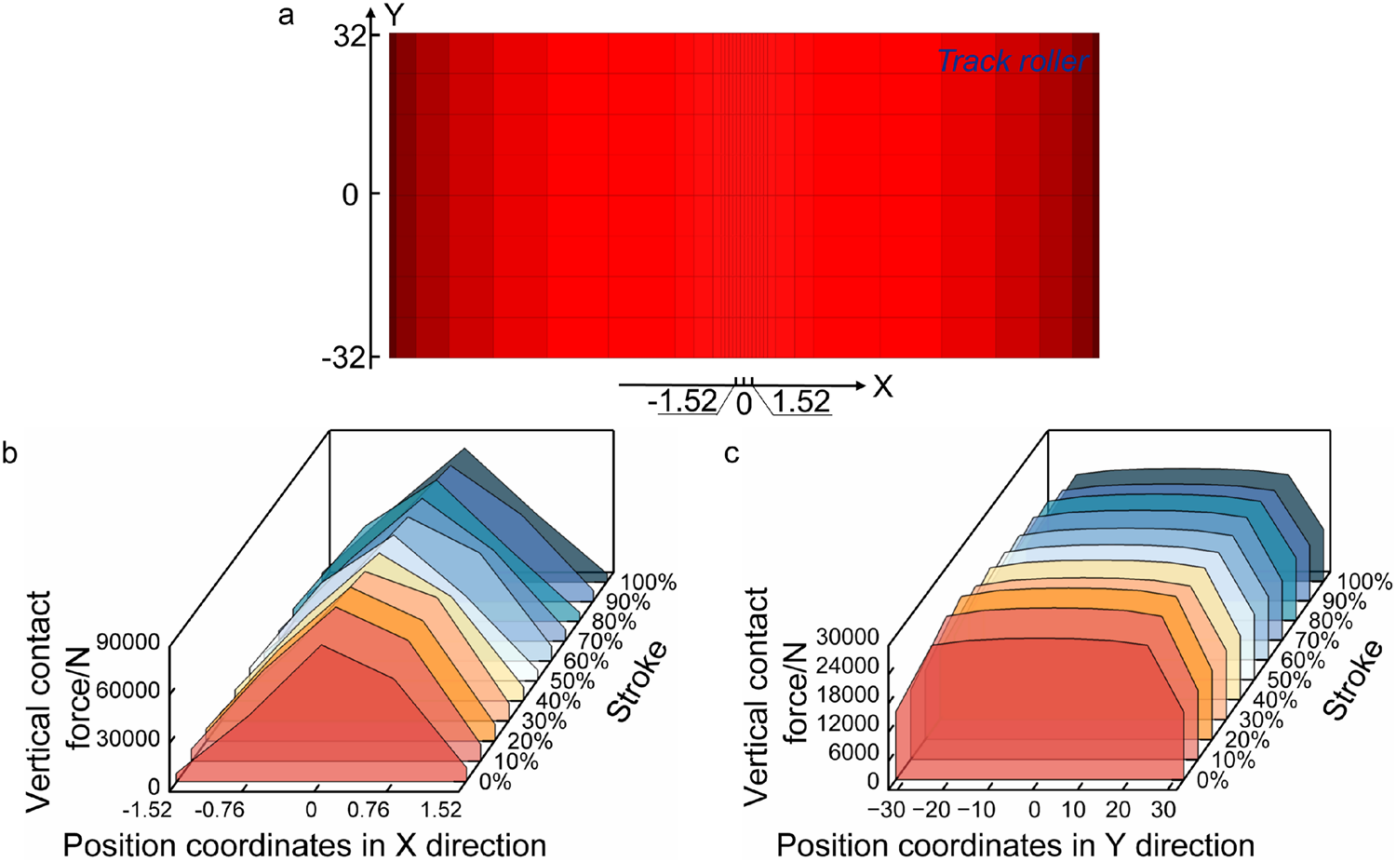
Vertical contact forces of the track roller at different stroke. (**a**) Sampling position coordinate diagram. (**b**) Vertical contact force in X direction. (**c**) Vertical contact force in Y direction.

To evaluate the adaptability of the improved support devices to load eccentricity, the contact regions between the top surfaces of the slider/track roller and the rack guide track were selected as sampling node paths. According to the coordinate systems shown in Fig. 16a and Fig. 17a, the nodal vertical contact forces over the full rack stroke from 0% to 100% were extracted to obtain the load distributions along the X and Y axes, as illustrated in Fig. 16 and Fig. 17. The maximum eccentric load factor for improved support devices and original LRG support device at each stroke are summarized in Table 8.

**Table 8:** Maximum eccentric load factor of three support schemes.

Support schemes	Maximum eccentric load factor	
	X-axis	Y-axis
LRG support device	1.68	1.72
Sliding support device	1.65	1.18
Track roller support device	-	1.15

Due to the large difference in the contact area between different support devices and the guide track, such as the contact area length of the sliding support scheme is much larger than that of the track roller support scheme, the eccentric load factor is defined as a normalized metric within the contact region of each support device rather than as a direct quantitative indicator of local stress intensity. Fig. 16b indicates that load eccentricity still exists in the sliding support device along the X-direction. Combined with Table 8, the maximum eccentric load factor of sliding support device is 1.65. Nevertheless, owing to the substantially increased contact area, the corresponding von Mises stress remains at a relatively low level. For the track roller support device, Fig. 17b shows that the vertical contact forces of the track roller are primarily concentrated within the contact half-width of ±1.52 mm, with no evident load eccentricity. As shown in Fig. 16c and Fig. 17c, the vertical contact force distributions of both the slider and track roller along the Y-axis are relatively uniform, with maximum eccentric load factor of 1.18 and 1.15, respectively. It should be noted that, since the width of the rack guide track is smaller than that of the slider and the outer ring of the track roller, the vertical loads near the edges of the contact interfaces are relatively lower than those in the central contact region.

**Fig. 18:**
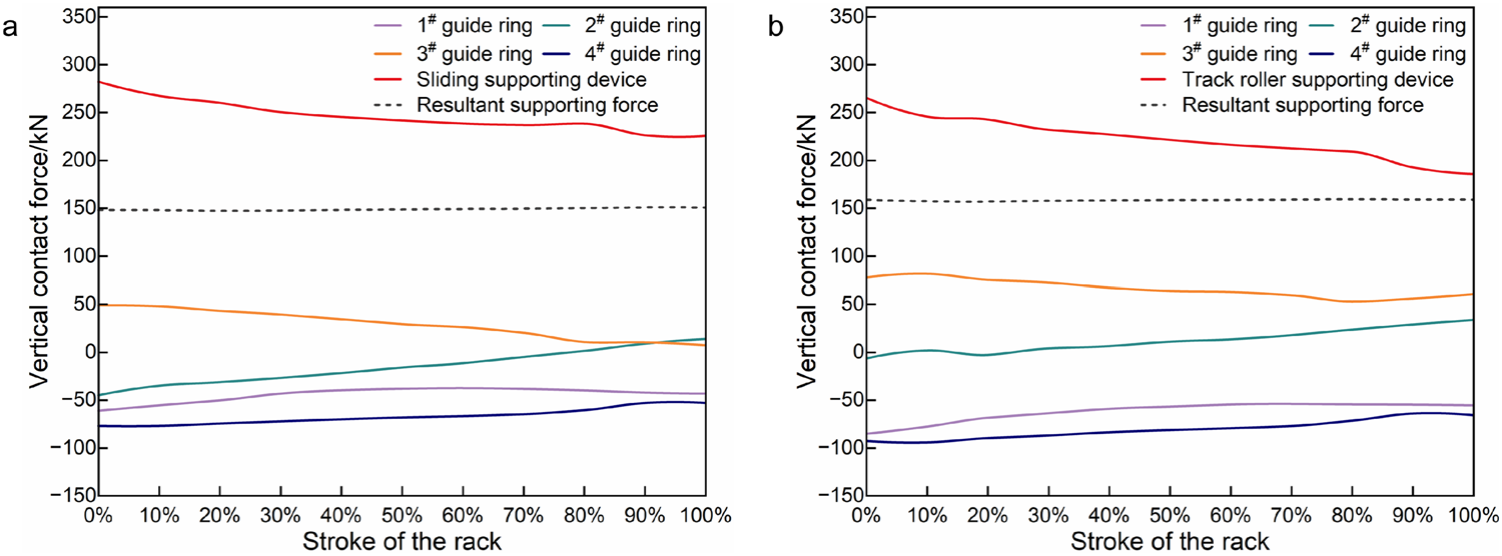
Supporting forces of the improved support devices and guide rings. (**a**) Sliding support device. (**b**) Track roller support device.

The vertical supporting forces acting on the rack from the support devices and the guide rings under the two improved support schemes were extracted, as shown in Fig. 18. As illustrated in Fig. 18a, the sliding support device bears most of the vertical load, with the average supporting force of 246.66 kN, while the supporting force provided by the guide rings ranges from −77.23 kN to 49.07 kN. Fig. 18b indicates that the track roller support device likewise carries the primary vertical load, with the average supporting force of 222.81 kN, and the guide ring supporting force ranging from −94.09 kN to 81.67 kN. Based on Fig. 11 and Fig.18, a comprehensive comparison of the ranges of supporting forces provided by the guide rings and the support devices under different schemes is summarized in Table 9.

**Table 9:** Comparison of supporting force of different schemes.

Support schemes	LRG	Sliding	Track roller
Average rack resultant supporting force/kN	137.63	148.93	158.41
Support device supporting force range/kN	[170.28, 241.33]	[225.61, 282.15]	[185.85, 265.38]
Guide ring supporting force range/kN	[−126.80, 110.68]	[−77.23, 49.07]	[−94.09, 81.67]

As shown in Table 9, the average rack resultant vertical supporting force under the LRG support scheme is 137.63 kN, whereas higher values are obtained for the sliding and track roller support schemes, reaching 148.93 kN and 158.41 kN, respectively. The differences in vertical supporting reactions among the three schemes can be attributed to their distinct frictional characteristics and constraint stiffness. Compared with the original LRG support device, the range of supporting forces sustained by the guide rings in both the sliding and track roller support schemes are reduced by 46.82% and 25.99%, respectively. Correspondingly, the range of supporting force sustained by both the sliding and track roller support schemes is higher than LRG support device. This indicates that the flexural deformation of the rack is effectively mitigated, and the vertical loads are primarily carried by the improved support devices, thereby substantially reducing the unintended load-bearing contribution of the guide rings. Consequently, the two improved support devices not only reduce the excessive local stresses responsible for roller crushing failure, but also eliminate the abnormal wear of the guide rings, thereby significantly enhancing the reliability and service life of the rack and pinion hydraulic actuator.

### Engineering feasibility and maintainability assessment

In Section "Validation of the effectiveness of the improved support device", the effectiveness of the sliding support device and track roller support device has been validated through FEA. Nevertheless, their practical engineering application in heavy-duty rack and pinion hydraulic actuators requires comprehensive consideration of manufacturing feasibility and maintenance characteristics.

Regarding manufacturing feasibility, the sliding support device features a relatively simple configuration. The CuAl10Ni slider requires standard CNC milling followed by surface grinding to achieve the required flatness. The PTFE pad can be produced via compression molding or extrusion forming. Since the slider transmits loads through surface contact, its load-bearing capacity primarily depends on effective contact area and overall stiffness matching, with relatively moderate tolerance requirements. In contrast, the track roller support device consists of multiple precision components, including the outer ring, inner ring, cylindrical rollers, retaining rings, and pin shaft.

The outer and inner rings require high-precision machining and heat treatment to ensure surface hardness and dimensional stability, while the rollers must satisfy strict geometric tolerances to guarantee proper load sharing. Moreover, assembly accuracy significantly impacts internal clearance and load distribution. Consequently, track roller support device demands higher manufacturing precision and quality control than sliding support scheme.

In terms of service performance and maintainability, the sliding support operates under sliding contact. While the enlarged contact area effectively reduces contact stress, progressive wear may still occur during long-term operation, requiring periodic inspection and replacement of wear components. By comparison, the track roller support device adopts rolling contact, which reduces friction losses and slows wear under proper lubrication conditions. Nevertheless, its reliability is more sensitive to installation alignment, with relatively higher maintenance complexity.

## Conclusion

This study presents a systematic investigation into the failure mechanisms of an LRG support device used in a heavy-duty rack and pinion hydraulic actuator, and proposes two improved support schemes validated through finite element analysis. The main conclusions and contributions are summarized as follows:

The underlying failure mechanisms associated with roller crushing and guide ring wear under heavy-duty operating conditions are elucidated in this study, which have not been systematically addressed in previous research. A high-fidelity finite element model of rack and pinion hydraulic actuator and LRG support device was established to capture the complex contact interactions and load transfer paths. The novelty of this work lies in revealing the coupled effects of rack pinion meshing force characteristics and rack flexural deformation on load non-uniformity in both longitudinal and transverse directions, providing a mechanical explanation for the observed failure.

The finite element analysis reveals that the failure of the original LRG support device is governed by severe load eccentricity, with the maximum von Mises stress reaching 2909.17 MPa at the end rollers, far exceeding the allowable stress of GCr15. This stress concentration is attributed to the combined effects of the vertical component of the rack pinion meshing force and the flexural deformation of the rack, which together induce non-uniform load distribution along both the longitudinal and transverse directions. Additionally, the irrational load transfer path causes the guide rings to sustain unintended vertical loads, accounting for the observed abnormal wear. These findings clarify the failure mechanisms and highlight the limitations of conventional LRG support device under heavy-duty operating conditions.

Two improved support devices, namely the sliding support and track roller support, were designed based on mechanical analysis and engineering constraints. The sliding support device converts discrete point/line contact into surface contact, significantly increasing the effective contact area and reducing the maximum von Mises stress to 65.83 MPa, well below the allowable stress of CuAl10Ni. The track roller support device, featuring a thick-walled outer ring and double-row cylindrical rollers, exhibits superior resistance to eccentric loading. The maximum stress is limited to 1050.28 MPa, remaining below the allowable stress of GCr15. Both improved support schemes effectively reconstruct a rational load transfer path, reducing the unintended load on guide rings by 46.82% and 25.99%, respectively, while ensuring that the vertical loads are primarily carried by the support devices.

The sliding support device offers advantages in structural simplicity, manufacturability, and maintenance convenience, whereas the track roller support device provides superior load distribution characteristics but involves higher manufacturing complexity and stricter assembly requirements. Both improved support devices provide practical engineering references for enhancing the support structures of rack and pinion hydraulic actuators in large-scale stamping equipment, forming machinery, and special-purpose actuators.

Future research should address these limitations by investigating the dynamic response and fatigue life of the proposed support devices under cyclic and impact loading conditions. In addition, experimental validation through prototype testing and long-term field trials is necessary to further verify the accuracy of the numerical analysis.

## Data Availability

The data that support the findings of this study are available from the corresponding author upon reasonable request.
